# ScanIndel: a hybrid framework for indel detection via gapped alignment, split reads and *de novo* assembly

**DOI:** 10.1186/s13073-015-0251-2

**Published:** 2015-12-07

**Authors:** Rendong Yang, Andrew C. Nelson, Christine Henzler, Bharat Thyagarajan, Kevin A. T. Silverstein

**Affiliations:** Supercomputing Institute for Advanced Computational Research, University of Minnesota, 117 Pleasant St. SE, RM 541, Minneapolis, MN 55455 USA; Department of Laboratory Medicine and Pathology, University of Minnesota, Minneapolis, MN 55455 USA

## Abstract

Comprehensive identification of insertions/deletions (indels) across the full size spectrum from second generation sequencing is challenging due to the relatively short read length inherent in the technology. Different indel calling methods exist but are limited in detection to specific sizes with varying accuracy and resolution. We present ScanIndel, an integrated framework for detecting indels with multiple heuristics including gapped alignment, split reads and *de novo* assembly. Using simulation data, we demonstrate ScanIndel’s superior sensitivity and specificity relative to several state-of-the-art indel callers across various coverage levels and indel sizes. ScanIndel yields higher predictive accuracy with lower computational cost compared with existing tools for both targeted resequencing data from tumor specimens and high coverage whole-genome sequencing data from the human NIST standard NA12878. Thus, we anticipate ScanIndel will improve indel analysis in both clinical and research settings. ScanIndel is implemented in Python, and is freely available for academic use at https://github.com/cauyrd/ScanIndel.

**Electronic supplementary material:**

The online version of this article (doi:10.1186/s13073-015-0251-2) contains supplementary material, which is available to authorized users.

## Background

Indel is the general term that may refer to an insertion or deletion of nucleotides in genomic DNA. Short indels (e.g., ≤10 bp) are the second most common type of polymorphism and long indels (e.g., >1 kb) are the most common structural variations (SVs) [1]. Detection of indels based on next generation sequencing (NGS) technologies is becoming more common [2], and current approaches include gapped alignment, split reads and *de novo* assembly [3].

Gapped alignment-based indel detection tools require interpretation of the alignment results from a gapped aligner such as BWA [4] in order to infer the presence of an indel [5]. Most of the commonly used variant detection programs, such as the GATK UnifiedGenotyper [6] and FreeBayes [7], belong to this category. A major drawback of these methods is the requirement that indels should be completely contained within a read and correctly identified during the initial read mapping step (reported as ‘I’ for insertion and ‘D’ for deletion in the CIGAR string [8]). This is sufficient for small indel detection, but is problematic for identifying indels that are longer than 15 % of the read length. In the long indel case, supporting reads will often contain too few bases that match the reference and therefore fail to map; or the supporting reads may have one end map well to the reference genome but the rest of the bases after the indel get trimmed or soft-clipped by the NGS aligner [9]. Split read methods (e.g., Pindel [10]) are designed to re-align soft-clipped reads to facilitate the identification of medium-sized indels, but it remains a challenge for these methods to distinguish low frequency true indel events from false-positive calls due to alignment errors. *De novo* assembly has been used to identify indels larger than the read length. For example, GATK HaplotypeCaller, Platypus [11] and Scalpel [12] employ localized or micro-assembly strategies and FermiKit [13] performs whole genome assembly for variant detection. Even though *de novo* assembly potentially can identify insertions of any size, it requires significant computational resources.

None of the existing methods are able to detect the full size spectrum of indels. We believe a hybrid approach that integrates multiple signals from all three sources (gapped alignment, split reads and *de novo* assembly) allows for more sensitive indel discovery than methods examining merely one or two signals. Based on this concept, we developed the **S**oft **C**lipping **An**alyzer for **Indel**s (ScanIndel). Our framework scans the initial mapping file from a gapped NGS aligner and refines the alignment of the soft-clipped reads meeting tiered criteria. Next, *de novo* assembly is performed for the selected soft-clipped reads and unmapped reads. Subsequent to the re-alignment and assembly, we have applied a Bayesian haplotype-based variant caller to detect indels. We present the results of ScanIndel analysis on (1) simulated data, (2) clinical data from targeted amplicon sequencing of tumor samples, and (3) human National Institute of Standards and Technology (NIST) standard NA12878 individual high coverage whole genome sequencing data. We compare the performance of ScanIndel with other existing tools for each data set.

## Methods

### Overview of ScanIndel

**Fig. 1:**
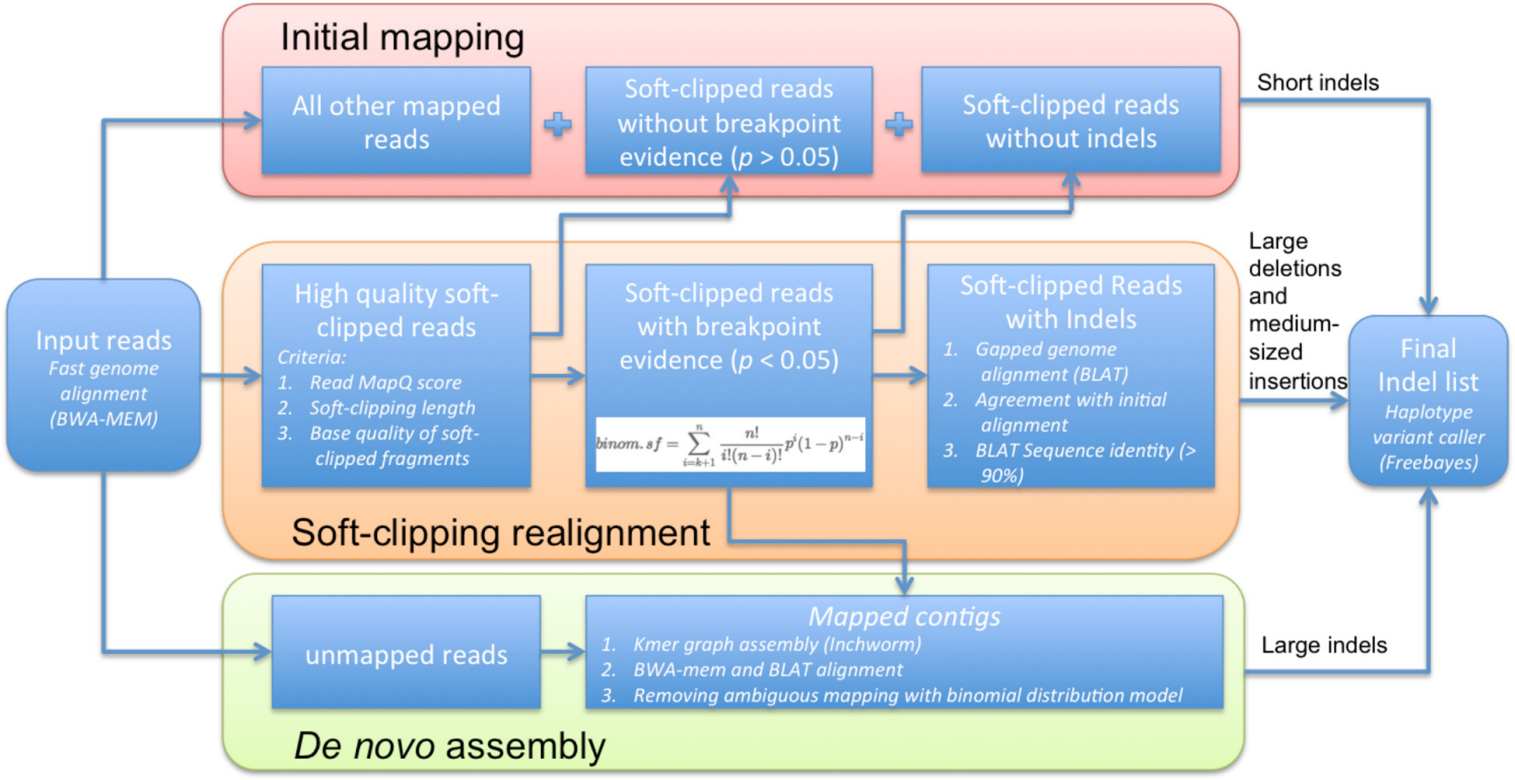
The ScanIndel workflow. ScanIndel aligns the raw read FASTQ files with a gapped NGS aligner (BWA-MEM) to detect short indels according to the initial mapping results. Soft-clipped reads with breakpoint evidence support are extracted for BLAT re-alignment to refine the CIGAR and genomic positions. Those re-aligned soft-clipped reads help to identify large deletions and medium-sized insertions. Meanwhile, ScanIndel carries out *de novo* assembly with the Inchworm assembler from Trinity for unmapped reads and BLAT realigned soft-clipped reads to detect large indels. All individual calling sets are merged by vcfcombine (from vcflib) to get one final VCF output containing all indel predictions

Sequence data are analyzed in a stepwise manner (Fig. 1). Input short reads are first aligned with a gapped NGS aligner. We used BWA-MEM [14] with default parameters as the raw read aligner, since BWA-MEM supports long-read and split-read alignment. Although BWA-MEM is used in this study, it can be replaced by another aligner that supports soft clipping and generates SAM or BAM output. After initial mapping to a suitable reference, the short reads are classified as three types: high quality soft-clipped reads, unmapped reads and all other mapped reads. We define high quality soft-clipped reads based on their mappability and length and the base quality of their soft-clipped fragments. Our hypothesis is that the high quality soft-clipped reads might either completely contain medium-sized indels or span the breakpoints of large indels. We next employ a binominal distribution to evaluate the significance of observing such soft-clipped reads caused by the presence of indels. Only soft-clipped reads with inferred breakpoint evidence are re-aligned using BLAT [15], which takes into account larger gaps than a NGS aligner to allow the precise identification of large deletions and medium-sized insertions covered by short reads. Simultaneously, *de novo* assembly is performed using the Inchworm assembler [16] for those putative breakpoint-covering soft-clipped reads together with unmapped reads, with the aim of constructing possible contigs that contain large indels that are longer than the soft-clipped fragments, especially for long novel sequence insertions. Finally, indels are collectively detected by FreeBayes, a Bayesian haplotype-based variant caller, from all three branches: initial raw read alignment that mainly accounts for short indels (e.g., ≤10 bp); soft-clipping realignment which reveals large deletions and medium-sized insertions (e.g., >10 bp but shorter than the read length) and *de novo* assembly which reports large indels (e.g., longer than the read length).

We describe the ScanIndel algorithm in detail below.

### Input data

ScanIndel accepts two types of input data: raw fastq files of short reads or a BAM file generated by a short read aligner with soft-clipping. If the input data are fastq files, ScanIndel utilizes BWA-MEM, mapping the reads first to generate a sorted and indexed BAM file for the next step. If the user inputs a BAM file, ScanIndel will sort and index it using SAMtools for further analysis.

### Identification of candidate soft-clipped reads

ScanIndel sorts all reads into three groups: unmapped reads, high quality soft-clipped reads and all remaining mapped reads from the initial mapping. Soft-clipped reads are coded as ‘S’ in their CIGAR string in the BAM file. Among them, high quality soft-clipped reads are defined with the following criteria: (i) read mapping quality (denoted by MAPQ in BAM) greater than a user-specified value (in practice, MAPQ ≥ 1); (ii) fraction of the soft-clipping part (in practice, ≥20 % of read length); (iii) proportion of high sequencing quality (in practice, minimum Q20) of soft-clipped bases (in practice, ≥80 %). With those filters, we try to exclude the reads that are soft-clipped due to bad sequencing quality or ambiguous alignment and only keep the reads with a long soft-clipped part that may suggest the presence of an indel within it.

### Breakpoint prediction

After collecting the high quality soft-clipped reads, we continue to filter those reads by examining if they have breakpoint evidence support. If a soft-clipped read contains the breakpoint(s) of an indel, we anticipate seeing a cluster of soft-clipped reads mapped to the same location around the breakpoints. Otherwise, it is not likely that the soft clipping was generated due to an indel. We calculate the probability (*P*) of a high quality soft-clipped read supported by breakpoint evidence with the following survival function of the binominal distribution:

1 $$ P={\displaystyle {\sum}_{i=k+1}^n}\frac{n!}{i!\left(n-i\right)!}{q}^i{\left(1-q\right)}^{n-i} $$

where *k* is the number of observed soft-clipped reads at a putative breakpoint, *n* is the total number of mapped reads at that locus and *q* is a user-specified heterogeneity factor (in practice, *q* = 0.1). We retain the high quality soft-clipped reads with breakpoint evidence (*P* < 0.05) for further realignment.

### Realignment of soft-clipped reads with breakpoint evidence

For each soft-clipped read with breakpoint evidence, BLAT is used to remap the read sequence to the reference genome. The multiple-hit results are first sorted by BLAT score as defined in the web-based UCSC BLAT (http://genome.ucsc.edu/FAQ/FAQblat.html). We accept the BLAT alignment only if the top ranked hit has a BLAT score > 30 with sequence identity > 90 %. The CIGAR string of the BLAT alignment is calculated using the method proposed by Heng Li (https://github.com/lh3/samtools-legacy/blob/master/misc/psl2sam.pl). Large deletions and medium-sized insertions that originally produced the BWA CIGAR string as soft clipping can be revealed by the calculation of the BLAT CIGAR string. Finally, we replace the BWA alignment of the soft-clipped reads with its BLAT alignment as described below:
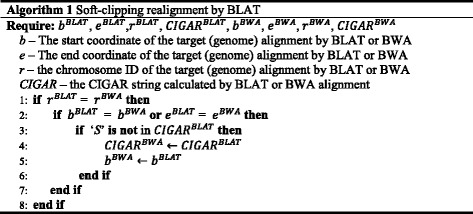


### *De novo* assembly of soft-clipped reads with breakpoint evidence and unmapped reads

The soft-clipped reads used for BLAT realignment and the unmapped reads from BWA alignment are assembled into contigs with the Inchworm algorithm, a part of the Trinity transcriptome assembler, by setting the K-mer = 25 and the minimal contig length to be at least one base longer than the read length. Each assembled contig is then aligned against the reference genome by applying a similar alignment procedure of short reads: BWA-MEM was used to carry out the initial alignment and soft-clipped contigs with breakpoint evidence were identified based on Eq. 1, and then re-aligned with BLAT to refine their CIGAR string and the leftmost mapping position in BAM file following Algorithm 1.

### Indel detection

ScanIndel produced two BAM files after soft-clipped read realignment and assembly. One BAM file is the alignment of all short reads from the NGS aligner with refined CIGAR and genomic positions using BLAT. The other BAM file is the alignment of assembled contigs after BWA and BLAT tiered mapping. Both BAM files are then sorted and indexed and passed as input to the haplotype-based variant caller, FreeBayes, for indel detection. Users can easily choose their own preferred variant caller (e.g., GATK) for variant calling. The short read BAM file mainly contributes to the identification of short indels, medium-sized insertions and large deletions. The contigs BAM file reports large indel predictions. Two VCF files are generated as output of FreeBayes for each of the BAM input files and they are merged into one final VCF file as ScanIndel output.

### Simulation data sets

We generated in silico data to evaluate our algorithm and compare it with several widely used indel detection methods. Human chromosome 20 (GRCh37/hg19 assembly) was used as the reference genome. Unannotated regions or assembly gaps, denoted by the letter ‘N’, were removed according to the UCSC Genome Browser gap track file. In general, indels were placed in a non-overlapping manner. First, the whole region was divided into 1-kb bins and then 2000 bins were randomly selected. Among the selected bins, the first half were used for placing deletions, one each with a size ranging from 1 bp to 1 kb and the second half were used to place insertions, again one each with a size ranging from 1 bp to 1 kb. The inserted sequences are randomly generated. Finally, the genome fasta file with the applied indels was created by svsim (https://github.com/mfranberg/svsim). We used wgsim (https://github.com/lh3/wgsim) to simulate sequencing reads from the generated target genome by setting the outer distance between the two ends to 500, standard deviation to 50, base error rate to 0.02 and the point mutation rate to 0.001 without allowing additional indel mutations.

### Evaluation metrics for indel calls

We considered the predicted indel calls for each algorithm to be true positives (TP) if the prediction met the following three criteria: (i) the predicted breakpoint was within 100 bp of the true breakpoint; (ii) the predicted size is equal to the true size; and (iii) the type of predicted mutation is identical to the true event. False positives (FP) are any prediction not meeting those criteria. False negatives are any real indel that are not identified by the detection tool. To assess the performance of each tool, we used precision (or positive predictive value) and recall (or sensitivity) as evaluation metrics as defined below:

$$ precision=TP/\left(TP+FP\right) $$

$$ recall=TP/\left(TP+FN\right) $$

### Analysis of targeted amplicon sequencing data

We tested our algorithm and the existing indel detection methods for sensitivity and specificity of clinically relevant indels in cancer with custom-designed amplicon NGS data. We first trimmed the 5’ adapter sequence AGACCAAGTCTCTGCTACCGTA from the left end and 3′ adapter sequence TGTAGAACCATGTCGTCAGTGT from the right end of the 300-bp reads using cutadapt v1.7 [17]. Then the trimmed 300-bp paired-end reads and 150-bp paired-end raw reads were aligned to the human reference hg19 with BWA-MEM v7.0.10. The sorted and indexed BAM file generated by BWA-MEM for each data set was used as input for ScanIndel, GATK, Pindel, Scalpel, Platypus and Delly. Raw fastq files were used as input for FermiKit. The short fragment nature of amplicon libraries and the uniform start-stop genomic positions characteristic of PCR products make the *de novo* assembly not well suited to analysis of amplicon data [18]. Therefore, ScanIndel was called with soft-clipping realignment only mode, which disabled the assembly step. Default parameters were used throughout for all software.

### Analysis of NA12878 whole genome sequencing data

Human NITS standard NA12878 was used to validate ScanIndel on whole genome sequencing (WGS) data. Raw fastq files were obtained from European Nucleotide Archives with the accession number ERA172924. Paired-end reads were aligned to the GRCh37 human reference using BWA-MEM v0.7.10 with default parameters and then duplicate reads were removed using Picard MarkDuplicates v1.68 (http://broadinstitute.github.io/picard/). We split the BAM file containing all reads by chromosome. Each smaller BAM file contains all mapped reads from only one chromosome and all unmapped reads without any mapping information in a large BAM file. All programs were called for each individual BAM file separately and predictions of each chromosome were merged into one final output file. Default settings were used for all tools except Scalpel, which was used with –-window 600 when running it for WGS data as recommended by the Scalpel manual (http://scalpel.sourceforge.net/manual.html). The running time of ScanIndel and Pindel is measured as the sum of running times for each chromosome and the peak memory is the maximum value of each individual run.

The Genome in a Bottle high-confidence call set was downloaded from the NCBI (ftp://ftp-trace.ncbi.nih.gov/giab/ftp/release/NA12878_HG001/). We removed all single-nucleotide polymorphism (SNP) calls from this data set. We extracted the predicted indels less than 20 bp from all programs we used and compared them against all called short indels (<20 bp) from the Genome in a Bottle truth set to measure the recall and precision of each method. The short tandem repeat (STR) region indels were identified if their genomic position has overlap with UCSC microsatellite track file measured by BEDTools v2.0 [19].

The large deletion reference data set used in our study was obtained by intersecting the data in Supplementary Table 1 from [20] with the data in Additional file 4 from [21]. All of those deletions were PCR validated and the large novel sequence insertion reference call set was obtained by extracting Cortex identified NA12878 sites from the 1000 Genomes Pilot 1 novel sequences file (ftp://ftp.1000genomes.ebi.ac.uk/vol1/ftp/pilot_data/paper_data_sets/companion_papers/mapping_structural_variation/union.2010_06.novelsequences.sites.vcf.gz). We further removed deletion and insertion calls in these two sets overlapping with potential mis-assembly regions used by SpeedSeq (https://github.com/cc2qe/speedseq#annotations). Additional file 1 shows those reference indel calls in BED format [22] with hg19 assembly.

**Table 1:** Indel detection (bp) by diffferent methods with 2 × 300-bp amplicon sequencing reads

Data set 1	Gene	Mutation	Tissue	Mean coverage	PCR/CE	ScanIndel	GATK	Pindel	Scalpel	Platypus	FermiKit	Delly
1-1	*CALR*	DEL	BM	46,913	52	52	52	NC	NC	NC	NC	NC
1-2	*EGFR*	DEL	FFPE	25,821	18	18	18	NC	NC	18	NC	NC
1-3	*KIT*	DEL	FFPE	12,990	6	6	6	NC	NC	6	NC	NC
1-4	*NPM1*	INS	BM	21,387	4	4	4	4	NC	NC	NC	NC
1-5	*FLT3*	INS	BM	4768	21	21	21	21	NC	NC	NC	NC
1-6	*FLT3*	INS	BM	11,510	26	27	NC	27	NC	NC	NC	NC
1-7	*FLT3*	INS	BM	26,747	49	51	NC	51	NC	NC	NC	NC
1-8	*FLT3*	INS	BM	28,991	38	39	NC	39	NC	NC	NC	NC
1-9	*FLT3*	INS	BM	26,734	90	93	NC	93	NC	NC	93	NC
1-10	*FLT3*	INS	BM	16,152	75	78	NC	78	NC	NC	NC	NC
1-11	*FLT3*	INS	BM	3528	32	33	33	33	NC	NC	NC	NC
1-12	*FLT3*	INS	BM	3851	51	54	54	54	NC	NC	NC	NC
1-13	*FLT3*	INS	BM	21,403	23	24	24	24	NC	NC	NC	NC
1-14	*FLT3*	INS	BM	3070	45	48	48	48	NC	NC	NC	NC
1-15	*FLT3*	INS	BM	4506	33	36	36	36	NC	NC	NC	NC
1-16	*FLT3*	NEG	BM	4471	NC	NC	NC	NC	NC	NC	NC	NC
1-17	*FLT3*	NEG	BM	4321	NC	NC	NC	NC	NC	NC	NC	NC
1-18	*FLT3*	NEG	BM	6219	NC	NC	NC	NC	NC	NC	NC	NC
1-19	*FLT3*	NEG	BM	5259	NC	NC	NC	NC	NC	NC	NC	NC
1-20	*FLT3*	NEG	BM	4443	NC	NC	NC	NC	NC	NC	NC	NC
1-21	*FLT3*	NEG	BM	5236	NC	NC	NC	NC	NC	NC	NC	NC
1-22	*FLT3*	NEG	BM	4443	NC	NC	NC	NC	NC	NC	NC	NC

*BM* bone marrow, *CE* capillary electrophoresis, *DEL* deletion, *FFPE* formalin-fixed paraffin-embedded, *INS* insertion, *NC* not called, *NEG* negative

## Results

### Calibrating the indel detection strategy used in the analysis

Simulation was used to calibrate ScanIndel, allowing unbiased estimation of the sensitivity and the limits of indel detection across different length scales. To keep runtimes short, we prepared the target genome based on human chromosome 20, which accounts for 2 % of the human genome but has a reasonably representative GC content, repeat content and gene density compared with the whole genome. Then, we randomly placed 1000 insertions and 1000 deletions throughout the targeted genome. The size of placed indels ranged evenly (one each size) from 1 bp to 1 kb. To assess the impact of coverage and read length on the algorithm performance, we generated two types of synthetic paired-end reads, 100 bp and 200 bp in length, and the targeted genome was ‘sequenced’ at 10×, 20× and 50× average coverage.

**Fig. 2:**
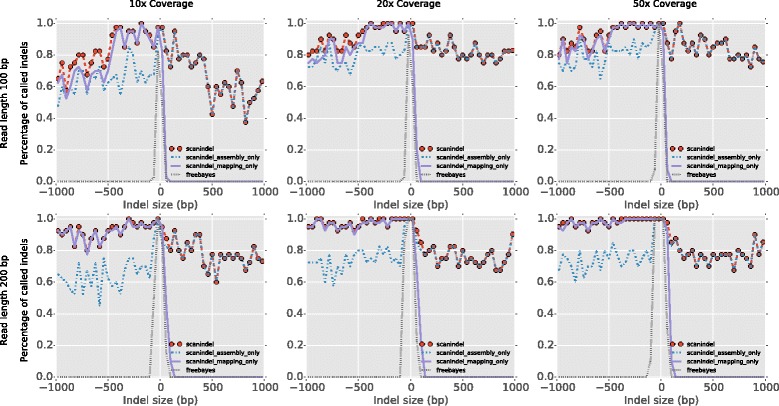
Effect of different strategies on indel detection. ScanIndel is executed by three modes: (1) BWA-MEM alignment + soft-clipping realignment + FreeBayes indel calling (labeled as ‘*scanindel_mapping_only*’); (2) BWA-MEM alignment + *de novo* assembly + FreeBayes indel calling (labeled as ‘*scanindel_assembly_only*’); and (3) complete ScanIndel procedures — BWA-MEM alignment + softclipping realignment + *de novo* assembly + FreeBayes indel calling (labeled as ‘*scanindel*’). In addition, FreeBayes indel calling directly from BWA-MEM alignment is tested as well (labeled as ‘*freebayes*’). Smoothed histograms (40-bp bins) showed the comparison on simulated short reads 100 bp and 200 bp in length under 10×, 20× and 50× mean coverage for detecting 1000 deletions and 1000 insertions ranged evenly in size from 1 bp to 1 kb

The simulation data were first aligned to the human reference genome hg19 using BWA-MEM. Next, we carried out indel analyses by going through each routine we proposed in the ScanIndel framework to measure the performance of each strategy. We first directly used FreeBayes for indel calling on the mapped raw reads. Second, we added the soft-clipping re-alignment step in the analysis without doing *de novo* assembly. Third, we performed *de novo* assembly only, without soft-clipping re-alignment. And lastly we employed the complete ScanIndel workflow on indel detection. As depicted in Fig. 2, FreeBayes reliably detected deletions as long as 40 bp and insertions up to 25 bp across different coverage depths when the read length was 100 bp. The number increased to 80 bp for deletions and 50 bp for insertions when the read length was 200 bp. Therefore, we infer that BWA-MEM tends to mark indels over 20 % of total read length as soft clipping. A recent study explored a range of common commercial and open source alignment tools (including BWA), and it reported that all those aligners failed to correctly align large indels, in agreement with our findings [23].

Next, we examined the detectability of indel size through utilizing soft-clipping re-alignment or *de novo* assembly, respectively. For the soft-clipping re-alignment only method, we found it can robustly detect large deletions (up to 1 kb) with high sensitivity (0.6 to 1). Higher coverage and longer read length increase the sensitivity for detecting deletions with lengths from 500 bp to 1 kb. For insertion detection, the soft-clipping re-alignment method performs better than raw read alignment. The maximum insertion size that can be detected goes up to around 50 bp when the read length is 100 bp and around 100 bp when the read length is 200 bp, respectively. However, soft-clipping re-alignment is still limited in detecting insertions that are longer than half of the total read length. We observed *de novo* assembly of soft-clipped and unmapped reads overcomes such limitation and is capable of detecting long insertions, even as long as 1 kb. Notably, we observed that the *de novo* assembly is capable of picking up some large deletions that are missed by soft-clipping realignment but is still less sensitive than soft-clipping realignment towards large deletion detection. Overall, when combining both soft-clipping realignment and *de novo* assembly approaches, we achieved the highest sensitivity for both insertions and deletions across the full spectrum of simulated indel sizes.

### Performance comparisons

We compared ScanIndel with six widely used indel detection tools (Pindel v0.2.5, GATK HaplotypeCaller v3.4.46, Platypus v0.8.1, Scalpel v0.4.1, Delly v0.6.7 and FermiKit v0.13) on our synthetic data sets. These algorithms were selected due to their ability to detect indels with base-pair resolution and to generate variants in VCF format, which is the standard to represent sequence variation. Pindel was the first split read-based indel detection tool to emerge, and was employed by the 1000 Genomes Project. The GATK HaplotypeCaller is the successor of UnifiedGenotyper, with a new local assembly feature for indel calling. Platypus is a haplotype-based variant caller that integrates both mapping and assembly approaches to enable long indel detection. Scalpel was recently developed as a *de novo* assembly-based indel caller and demonstrated substantial improvement over other popular indel tools such as SOAPindel. Delly is capable of detecting large deletions [24]. FermiKit is considered a better long insertion caller [13]. All programs were called using default parameters with minor adjustments and the analyses were based on BWA-MEM alignment in a manner similar to ScanIndel (Additional file 2).

**Fig. 3:**
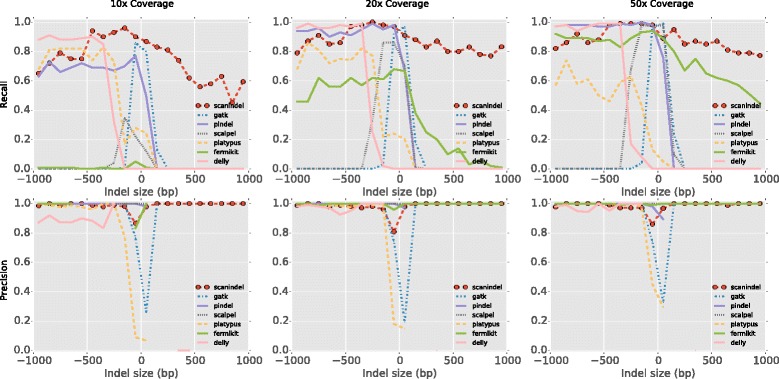
Performance comparison for indel detection with 100-bp simulated reads. Recall (*upper panels*) and precision (*lower panel*s) are evaluated for ScanIndel, GATK HaplotypeCaller, Pindel, Platypus, Scalpel, Delly and FermiKit. Smoothed histograms (100-bp bins) showed the comparison on simulated data of 10×, 20× and 50× mean coverage for detecting 1000 deletions and 1000 insertions, one each from the size range 1 bp to 1 kb. Precision is not calculated if a zero denominator (TP + FP = 0) is given by the method

We measured performance with recall (or sensitivity) and precision (or positive predictive value) to evaluate the ‘probability of calling a validated variant’ and the ‘probability that a called variant is correct’, respectively. We observed that ScanIndel was the only algorithm that detected all sizes of deletions and insertions across all coverage levels for both 100-bp and 200-bp reads (Fig. 3; Additional file 3). ScanIndel achieved the highest recall and precision of all of the methods tested when sequencing depth was only 10× in both read length cases, suggesting it reliably detected indels even in the low coverage scenario. ScanIndel, Delly and Pindel had the best performance in detecting large deletions (>500 bp), but Delly and Pindel showed limited power to detect large insertions (>100 bp). At 50× coverage, both ScanIndel and FermiKit detected large insertions. However, at lower coverage (10×), only ScanIndel could still reliably detect large insertions. GATK, Platypus and Scalpel demonstrated limited capability to detect large indels. In general, our results showed existing split read and local assembly technologies had reduced power in comparison with ScanIndel to detect large insertions and deletions (or both) when their sizes were over twice the read length. This suggests that the combination of split read and assembly enables better detection of long indels than either single mode.

### Detection of clinically actionable indels in tumor samples from targeted, amplicon-based sequencing data

To test ScanIndel on clinical data, we used it on a custom-designed amplicon NGS assay containing 93 amplicons spread over 21 genes (Additional file 4). Targeted sequencing of the customized cancer gene panel from 15 tumor specimens was performed by an Illumina MiSeq sequencer with 300-bp paired-end reads to an average depth of coverage over 3000×. Of these, 11 specimens were *FLT3* internal tandem duplication (ITD)-positive with insertions ranging from 21–90 bp, seven specimens were *FLT3* ITD-negative, one specimen was *CALR* 52 bp-deletion-positive, one specimen was *EGFR* 18 bp-deletion-positive, one specimen was *KIT* 6 bp-deletion-positive and one specimen was *NPM1* 4 bp-insertion-positive. All of the those mutations were orthogonally detected in the clinical laboratory by either PCR and capillary electrophoresis or outside laboratory testing performed in Clinical Laboratory Improvement Amendments (CLIA)-licensed laboratories. Additionally, we also sequenced specimens for a subset of *FLT3* ITD-positive and all other genes using the V2 Illumina chemistry with 150-bp paired-end reads (Additional file 5).

We applied ScanIndel on both 300-bp and 150-bp read data sets and compared it with other widely used NGS tools to test their performance on the 300-bp read data set (see Methods for details). The results achieved with each software tool are summarized in Table 1. Only ScanIndel detected all indels in these cases and the ScanIndel results were generally compatible with PCR and capillary electrophoresis. In contrast, existing tools performed poorly for either deletion or insertion or in both cases. Pindel successfully detected all *FLT3*-ITD insertions, which agreed with a previous study [25], but failed to detect all validated deletions in *CALR*, *EGFR* and *KIT*. GATK was able to detect all deletion cases but missed the longer insertions. Other tools either occasionally reported indels or had no predictions at all, suggesting that those general purpose analysis tools might not be suited for amplicon-based NGS assays.

ScanIndel achieved 100 % sensitivity and 100 % specificity for all *FLT3* insertion detection attempts with 300-bp reads (Table 1). When we inspected the *FLT3* indel detection for 150-bp reads, we noted that the 93-bp *FLT3* insertion was missed (Additional file 4). Although the choice of read length for insertion identification is a rather open-ended question; our results suggest that longer reads (e.g., 300 bp) will enable better identification of longer clinically actionable insertions, such as *FLT3* ITD. For all deletion cases we have tested, ScanIndel demonstrated 100 % sensitivity, including the largest 52-bp *CALR* deletion (Table 1; Additional file 4). Our method robustly detected deletions in both 300-bp and 150-bp reads, indicating it outperformed the existing clinical amplicon-based NGS data processing pipeline for large deletion detection [26], which is essential for accurate clinical diagnostics.

### Application to WGS (50×) of human individual NA12878

To assess the performance of ScanIndel on WGS data, we analyzed a well-studied HapMap sample NA12878. The 100-bp paired-end data with an average coverage of 50× were provided through Illumina’s Platinum genomes project. Genome in a Bottle Consortium has provided a high-confidence call set for sample NA12878, which includes SNPs and indels [27]. Most of the called indels are less than 20 bp (Figure S2a in Additional file 6).

We tested ScanIndel against the Genome in a Bottle call set by measuring its recall and precision on short indels and compared them with Pindel, Scalpel, Platypus and FermiKit. For short indels, we observed that ScanIndel achieved the highest sensitivity (over 90 %) and exhibited comparable precision with the existing tools (Figure S2b, c in Additional file 6). A major source of error in short indel detection is within STR structures [28]. Hence, we specifically compared the performance of ScanIndel with other tools in regions containing STRs. The Genome in a Bottle set contains 33,676 called indels from STRs. Additional file 7 displays the sensitivity and precision of short indel (<20 bp) detection at STR regions between methods. ScanIndel and FermiKit performed the best for predicting the true positives. Scalpel worked slightly better than the other tools for reducing the false positive rate.

To test the performance on large indels, we applied ScanIndel and the other tools tested in the simulation data to this sample and compared their predictions with two curated reference sets: (i) 138 validated deletions from the literature; and (ii) 105 previously predicted novel sequence insertions identified by the 1000 Genomes Project (see "Methods" for details).

**Fig. 4:**
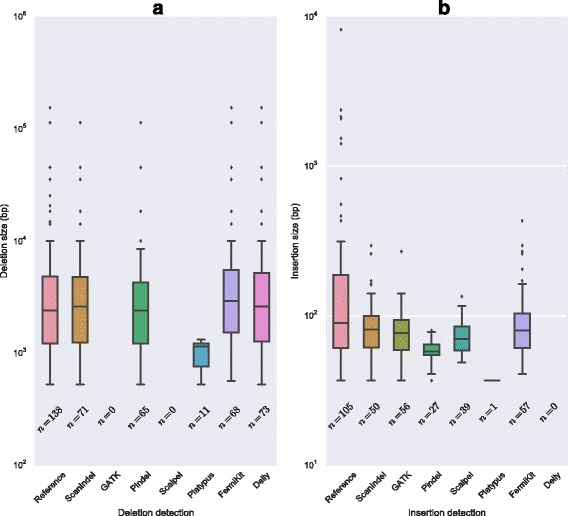
Performance comparison of large indel detection on NIST standard NA12878. **a** Validated large deletions (138) from the literature with sizes from 530–155,154 bp are used as a reference standard set. **b** Novel sequence insertions (105) previously identified by the 1000 Genomes Project with sizes from 37–8224 bp are used as reference standard

As shown in Fig. 4a, Delly predicted the highest number of expected deletions, which is not surprising since it was designed to detect large scale SVs. ScanIndel ranked second for large deletion detection and predicted almost as many as Delly, suggesting that ScanIndel was able to perform as well as SV detection tools for large sized deletions. Figure 4b shows the results of novel sequence insertion detection. ScanIndel detected 50 insertions, which was very close to the performance of the best predictor, FermiKit. Our results suggest that ScanIndel is capable of predicting longer insertions with similar performance as the true assembly-based methods which tend to be most efficient in the detection of large insertions. Taken together, ScanIndel generally outperformed the other tools when calling across the spectrum of both short and large indels.

**Fig. 5:**
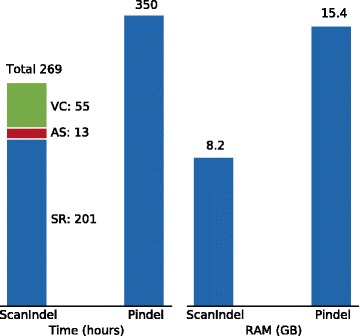
Time and peak memory used by ScanIndel and Pindel on NA12878 individual 50× WGS data. The run time of ScanIndel is counted in each module: split read re-alignment (*SR*), *de novo* assembly (*AS*) and variant calling (*VC*). All the measurements refer to the program itself, and do not include BWA-MEM alignment

Finally, we compared the run time and memory usage of our method with Pindel when analyzing this high coverage WGS data set using an 8-core Intel Xeon @ 2.66 GHz with 16 GB of memory. ScanIndel is mainly composed of three steps: soft-clipping realignment, assembly and variant calling. Figure 5 shows that the major part of the running time is spent on the soft-clipping realignment step. This was expected considering that BLAT, which was used for soft-clipping realignment, was time-consuming when the set of reads was large. However, with our heuristic algorithm, we limited BLAT realignment to only a small fraction of soft-clipped reads from the WGS data to significantly decrease the running time. As a result, ScanIndel spent 81 hours less than Pindel to complete the analysis. Notably, ScanIndel only required 8.2 GB peak memory, which was only half of Pindel’s memory cost. Taken together, we have shown ScanIndel is a fast and memory-efficient indel detection algorithm for a large-scale data set.

## Discussion

We introduced a new algorithm for detecting indels by integrating both mapping and assembly technologies. Our method is efficient in detecting medium-sized insertions and large deletions by heuristic re-mapping of soft-clipped reads produced by a short read aligner which may contain the breakpoints of longer indels. Our method employed a K-mer-based assembly algorithm to identify larger novel sequence insertions. Our testing on simulated and real data demonstrates that ScanIndel outperforms the existing split read- or assembly-based indel calling tools for accurately detecting indels across the whole size spectrum.

Widely used Burrows–Wheeler transform (BWT)-based short read aligners are poor at correctly mapping reads with large gaps. Although modifying default parameters, such as gap opening and extension penalty options in BWA, can improve indel detection, soft-clippings remain due to the existence of missed larger indels. Therefore, split read realignment without using BWT is necessary to allow reads to be globally mapped. ScanIndel employs BLAT to refine the alignment of split reads, since it is known that BLAT is much better than short read aligners at mapping sequences with gaps. The main drawback of BLAT is its poor speed of execution when the set of reads is large. Instead of realigning all split reads generated by a BWT aligner, ScanIndel applies a novel heuristic search method to create a small set of split reads with putative breakpoint information for BLAT mapping. This significantly decreases the total running time, and makes even high coverage WGS analyzable in a reasonable time.

Soft-clipped read realignment can theoretically find deletions of any size, but it has limited power to detect insertions owing to the short read length of current sequencing technologies. To extend the power of detectable mutations using short reads, ScanIndel employs an assembly strategy to detect large indels. Two major paradigms are used for existing assembly-based variant detection technologies. The first approach is to perform *de novo* whole-genome assembly of the reads and detect variations between the assembled contigs and the reference genome (e.g., Fermi [29]). The other, recently popular paradigm is to perform localized micro-assembly of soft-clipped reads and unmapped reads that are anchored by their mapped mate around specific regions of interest in a genome (e.g., Scalpel [12]). Although the global assembly paradigm has the potential to detect larger mutations, in practice it is less sensitive and more time consuming because a large proportion of reads used for assembly are not from candidate indels. In contrast, the local assembly paradigm is efficient to determine longer insertions, but is limited by the size (since completely unmapped read pairs are discarded which may be part of inserted sequences). ScanIndel incorporates a semi-global assembly strategy by collecting only soft-clipped reads with breakpoint evidence and all unmapped reads for assembly all at once. By this approach, only a small set of reads is processed to save running time without losing breakpoint information provided by soft-clipped reads.

Assembly algorithms are a crucial component of an indel detection pipeline. Despite the fact that many *de novo* assembly programs have been developed and are publicly available, they are designed for maximizing the coverage of the underlying genome sequence [30]. Therefore, all of these algorithms attempt to organize the sequencing reads into very long contigs (median length of 10–50 kb). This is substantially different from our goal to identify disjoint, medium length (median of 1 kb) sequences each of which contains the novel sequence insertion. To achieve this goal, we chose the Inchworm assembler [16] because it is fast and was originally designed for organizing reads into a suitable length contig to predict gene isoforms.

Amplicon-based targeted NGS assays are widely used to identify clinically actionable somatic alterations in cancer [31]. In our analysis, we observed that alignment-based variant callers such as GATK, Pindel and Platypus were able to detect some of the known indels from our amplicon data. However, assembly-based methods such as Scalpel and FermiKit did not work in our case, which is not surprising since analysis of targeted amplicon sequencing data presents some unique challenges in comparison with the analysis of random fragment sequencing data. Whereas reads from randomly fragmented DNA have arbitrary start positions, the reads from amplicon sequencing have fixed start positions that coincide with the amplicon boundaries. As a result, assembly will not extend amplicon reads into longer contigs. Because of the fundamentally different nature of the amplicon sequencing from whole genome and hybrid capture sequencing assays, it precludes the application of a variety of assembly-based indel detection algorithms commonly used for random fragment sequencing data [18].

Our simulation results have shown that split read methods perform better than assembly-based methods for larger deletion detection, while assembly methods are able to detect longer insertions than split read methods. Therefore, utilizing both strategies is essential to successfully detect indels of any size. To our knowledge, ScanIndel is the only method that has been specifically developed to perform both soft-clipping realignment and *de novo* assembly for indel detection. Our application of ScanIndel to targeted resequencing and WGS data has shown its success for both somatic and germline indel detection.

## Conclusions

We present ScanIndel as a robust method for detecting indels from targeted amplicon-based to WGS data. In particular, ScanIndel reliably detects medium-size indels and has comparable performance with existing methods for detecting very large indels. ScanIndel is capable of detecting indels across the full size spectrum with base-pair resolution. We anticipate ScanIndel will enable identification and elucidation of indels that are currently difficult to characterize.

## Additional files

Additional file 1: Table S1. A list of 138 validated deletions from the literature and 105 previously predicted novel sequence insertions identified by the 1000 Genomes Projects for human NIST standard NA12878. The genome coordinates are mapped to hg19. (XLSX 18 kb) Additional file 2: Table S2. Parameters used for Indel detection tools. (XLSX 25 kb) Additional file 3: Figure S1. Performance comparison for indel detection with 200-bp simulated reads. Recall (*upper panel*) and precision (*lower panel*) are evaluated for ScanIndel, GATK HaplotypeCaller, Pindel, Platypus, Scalpel, Delly and FermiKit. Smoothed histograms (100-bp bins) show the comparison on simulated data of 10×, 20× and 50× mean coverage for detecting 1000 deletions and 1000 insertions, one each from the size range 1 bp to 1 kb. Precision is not calculated if a zero denominator (TP + FP = 0) is given by the method. (PDF 26 kb) Additional file 4: Table S3. Targeted regions of cancer gene panel. The genome coordinates are mapped to hg19. (XLSX 11 kb) Additional file 5: Table S4. Indel detection by ScanIndel with 2 × 150-bp amplicon sequencing reads. (XLSX 44 kb) Additional file 6: Figure S2. Performance comparison of short indel detection on NIST standard NA12878. The Genome in a Bottle high-confidence indel call set is used for benchmarking. **a** Distribution of indel size of the truth set. The *black curve* is the fitted gamma distribution density estimation. **b**, **c** Calculated recall and precision of predicted short indels (<20 bp) by different programs against the truth set. (PDF 20 kb) Additional file 7: Figure S3. Performance comparison for indel detection from short tandem repeat region of the NA12878 sample. Recall and precision are evaluated for ScanIndel, Pindel, Platypus, Scalpel, Platypus and FermiKit against the called indels from the Genome in a Bottle benchmark set for indels less than 20 bp. (PDF 31 kb)

## Abbreviations

bp: base pair
BWA: Burrows–Wheeler Aligner
BWT: Burrows–Wheeler transform
FP: false positive
ITD: internal tandem duplication
kb: kilo base
NGS: next generation sequencing
NIST: National Institute of Standards and Technology
PCR: polymerase chain reaction
SNP: single-nucleotide polymorphism
STR: short tandem repeat
TP: true positive
WGS: whole genome sequencing
