# Supplementary figures and images for: ScanIndel: a hybrid framework for indel detection via gapped alignment, split reads and de novo assembly
Source: Genome Med. 2015 Dec 7;7:127. doi: 10.1186/s13073-015-0251-2 (PMC4671222; doi:10.1186/s13073-015-0251-2)

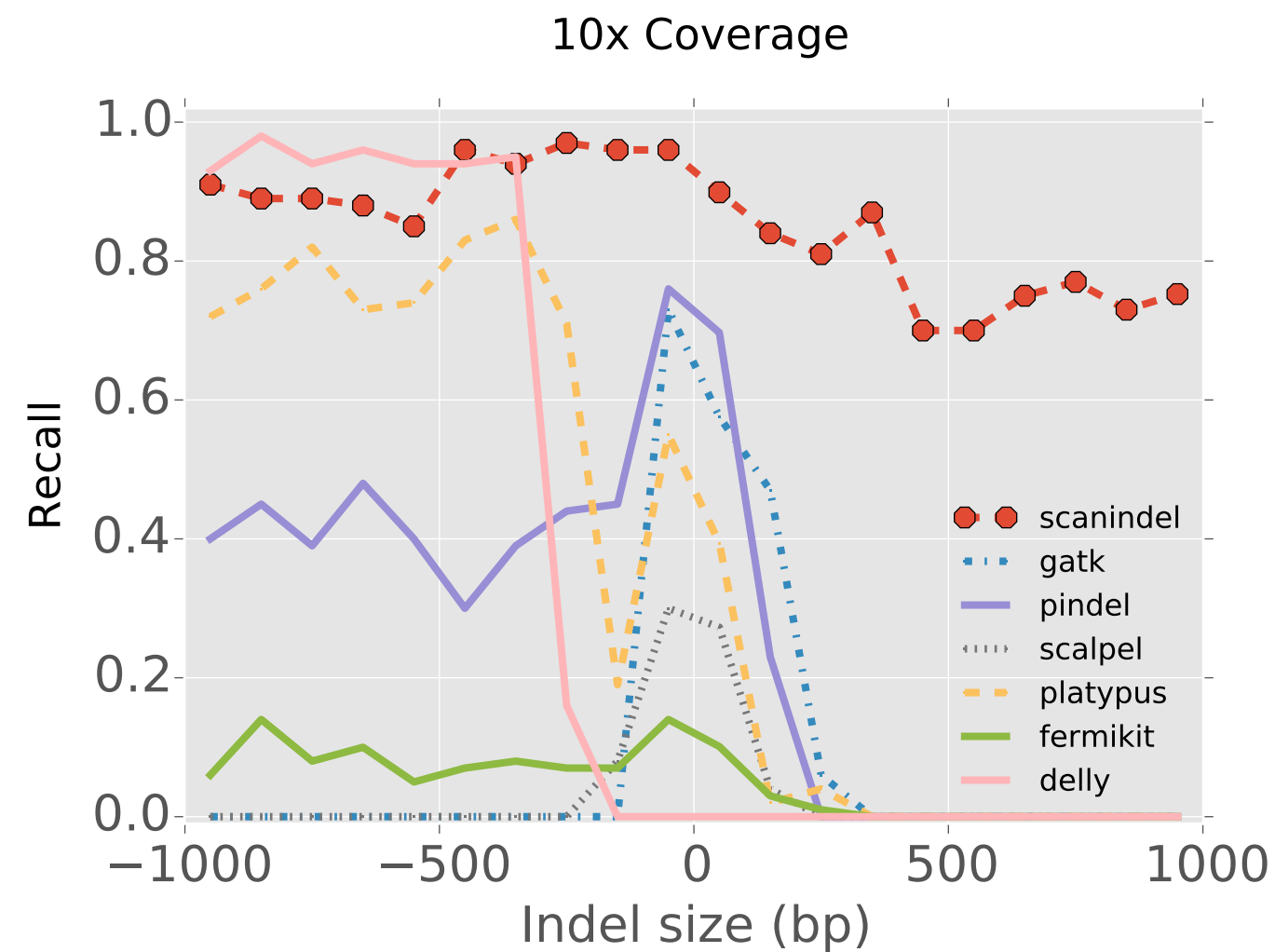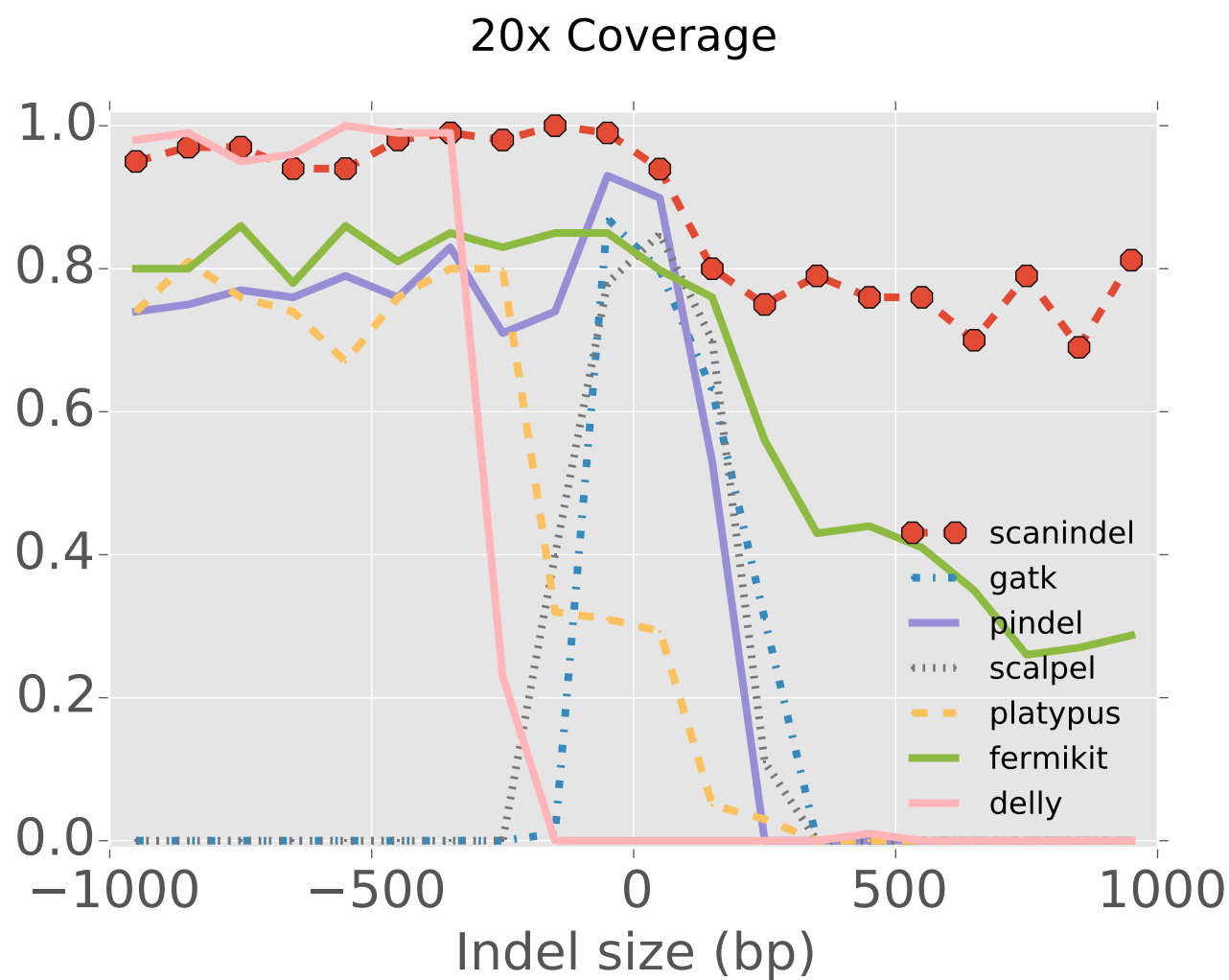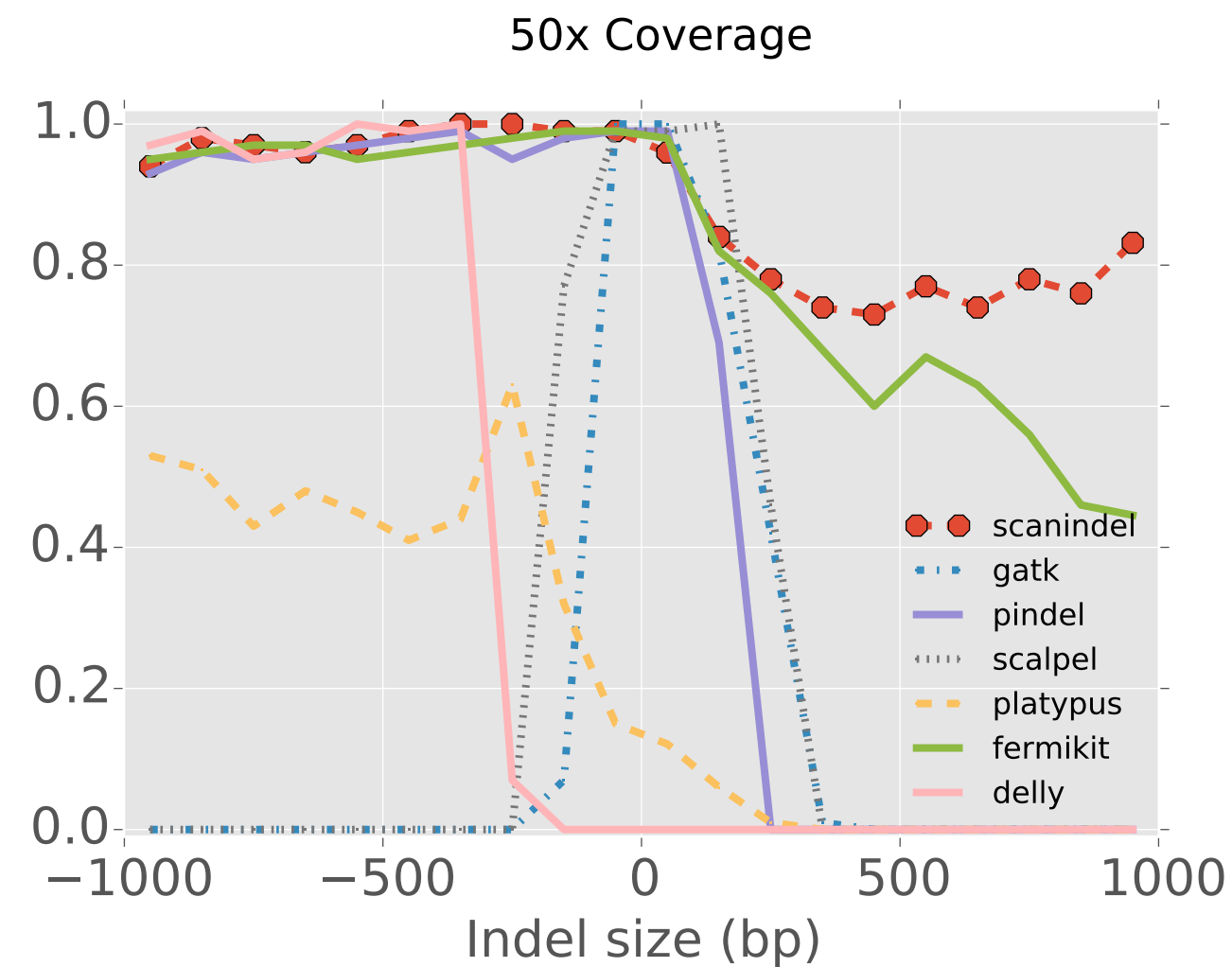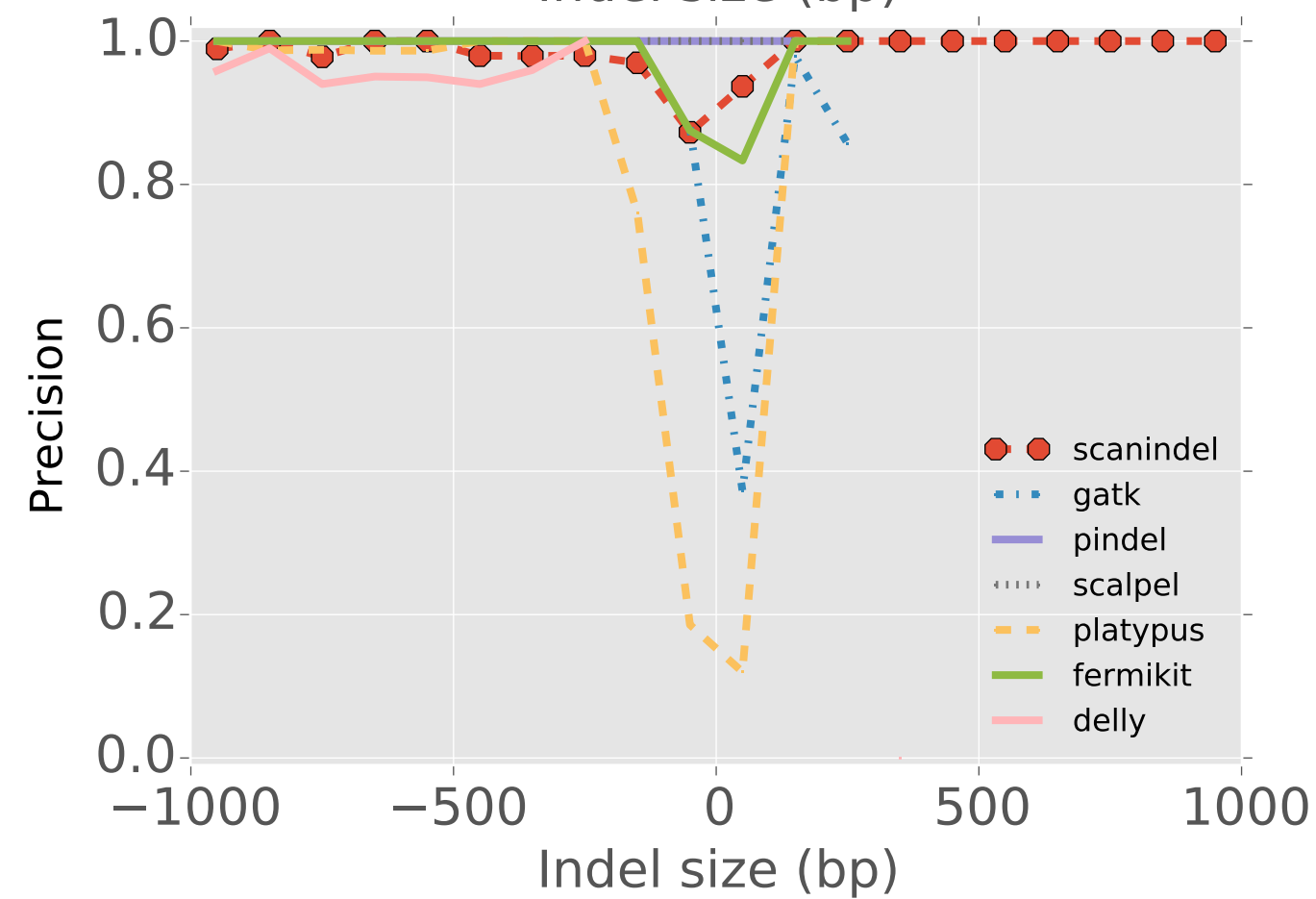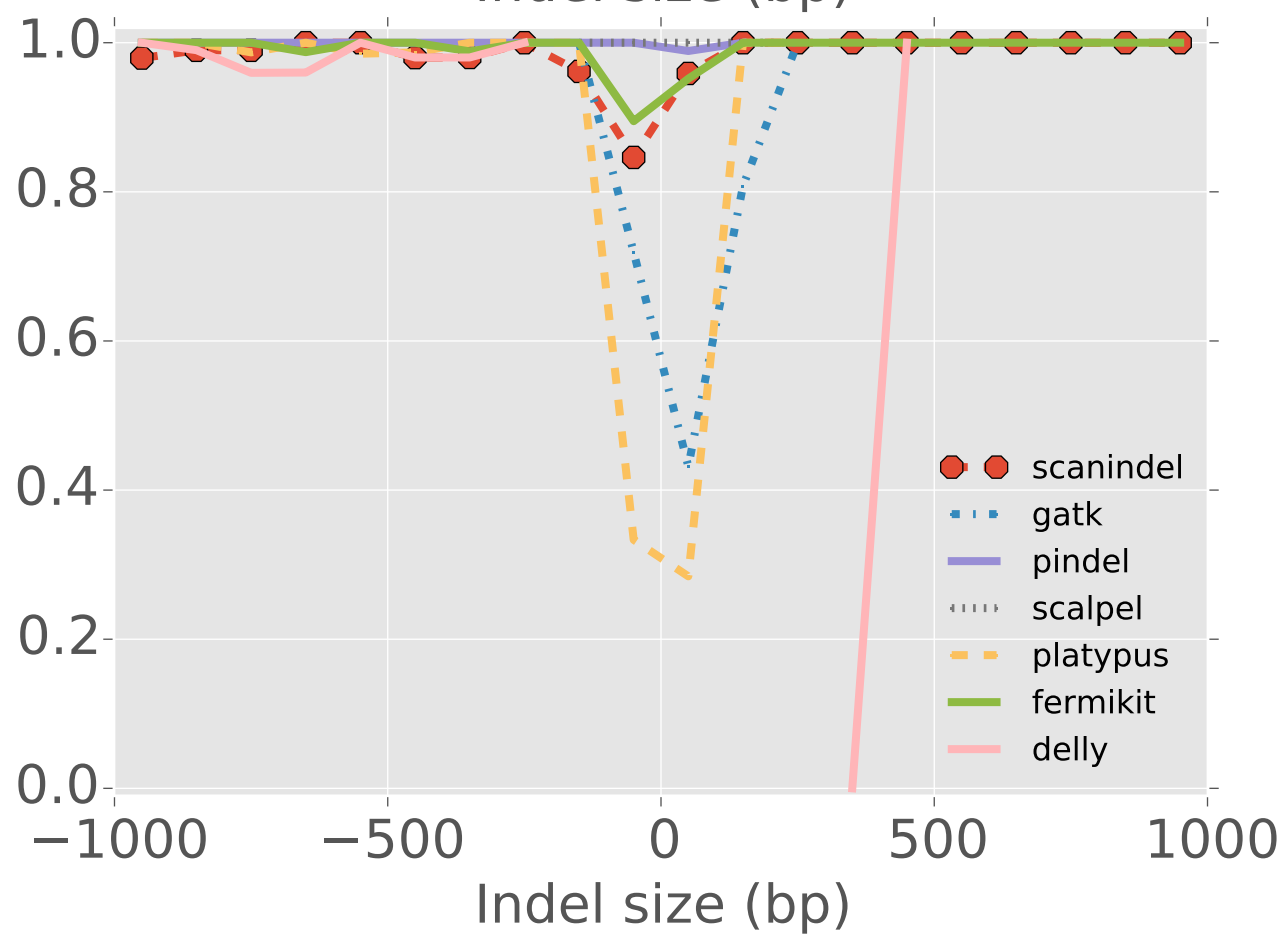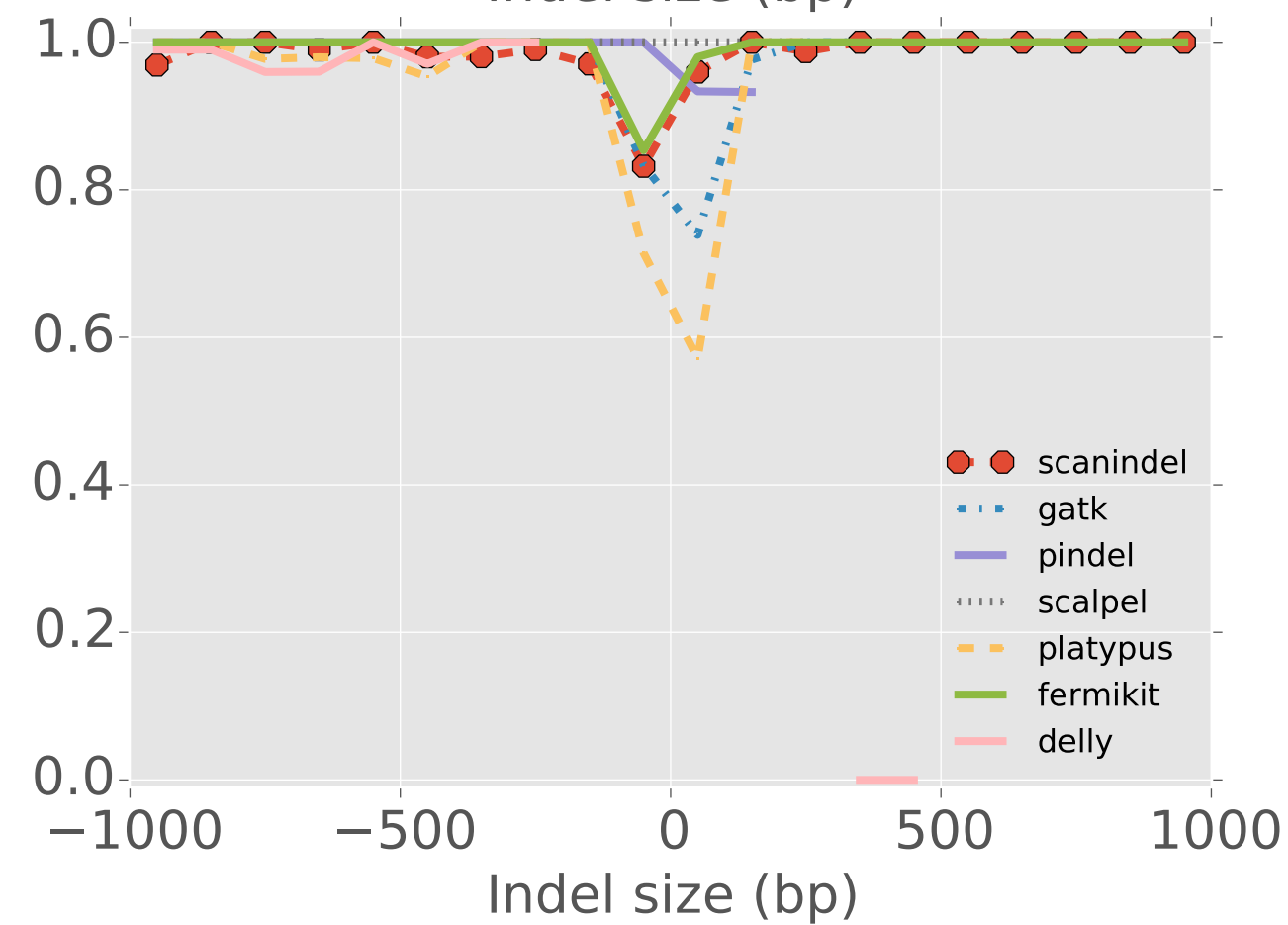

Supplement: Additional file 3: Figure S1. — Performance comparison for indel detection with 200-bp simulated reads. Recall (upper panel) and precision (lower panel) are evaluated for ScanIndel, GATK HaplotypeCaller, Pindel, Platypus, Scalpel, Delly and FermiKit. Smoothed histograms (100-bp bins) show the comparison on simulated data of 10×, 20× and 50× mean coverage for detecting 1000 deletions and 1000 insertions, one each from the size range 1 bp to 1 kb. Precision is not calculated if a zero denominator (TP + FP = 0) is given by the method. (PDF 26 kb) [file 13073_2015_251_MOESM3_ESM.pdf]

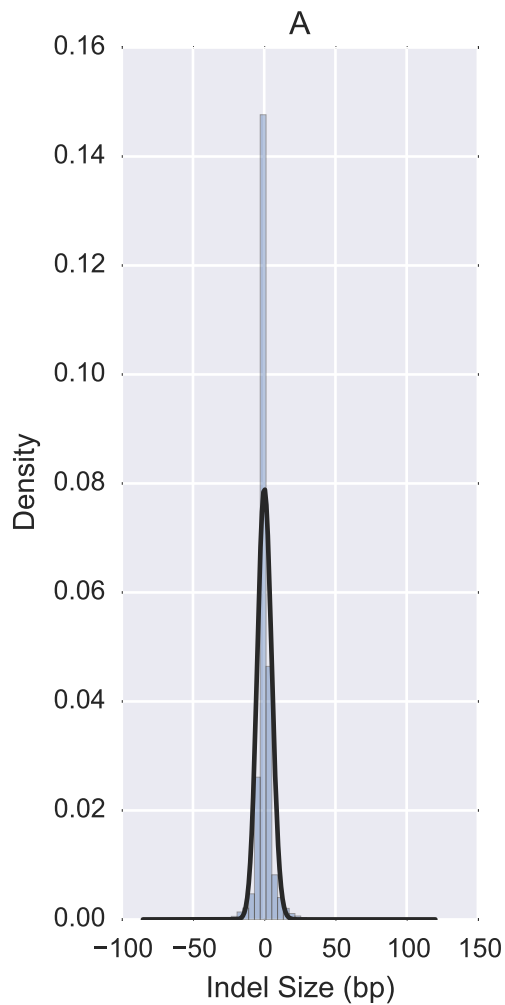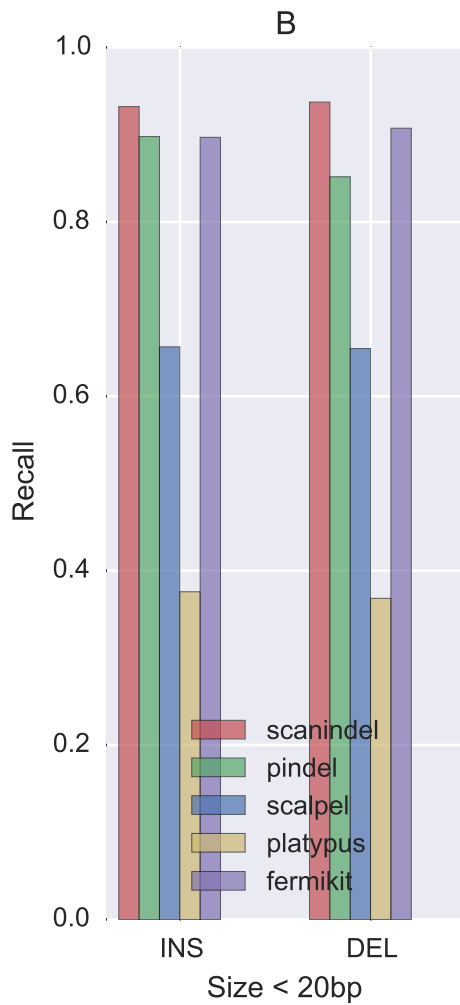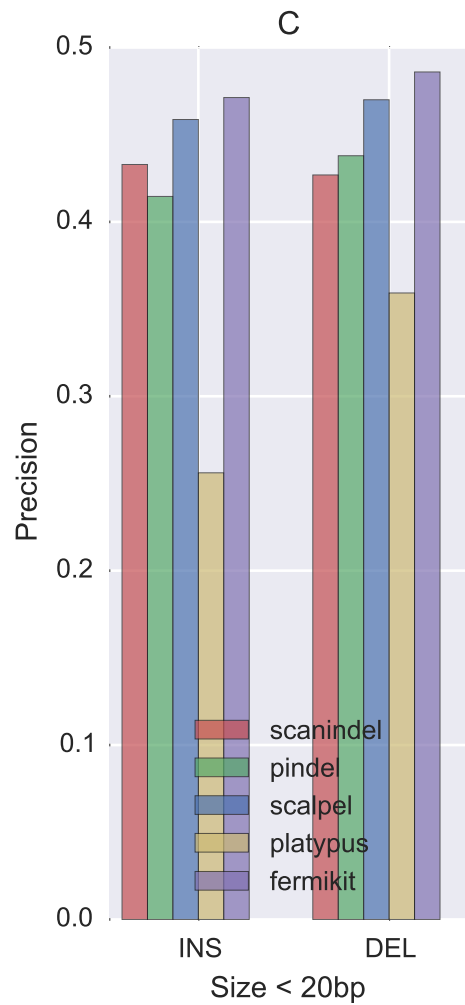

Supplement: Additional file 6: Figure S2. — Performance comparison of short indel detection on NIST standard NA12878. The Genome in a Bottle high-confidence indel call set is used for benchmarking. a Distribution of indel size of the truth set. The black curve is the fitted gamma distribution density estimation. b, c Calculated recall and precision of predicted short indels (<20 bp) by different programs against the truth set. (PDF 20 kb) [file 13073_2015_251_MOESM6_ESM.pdf]

## Sensitivity

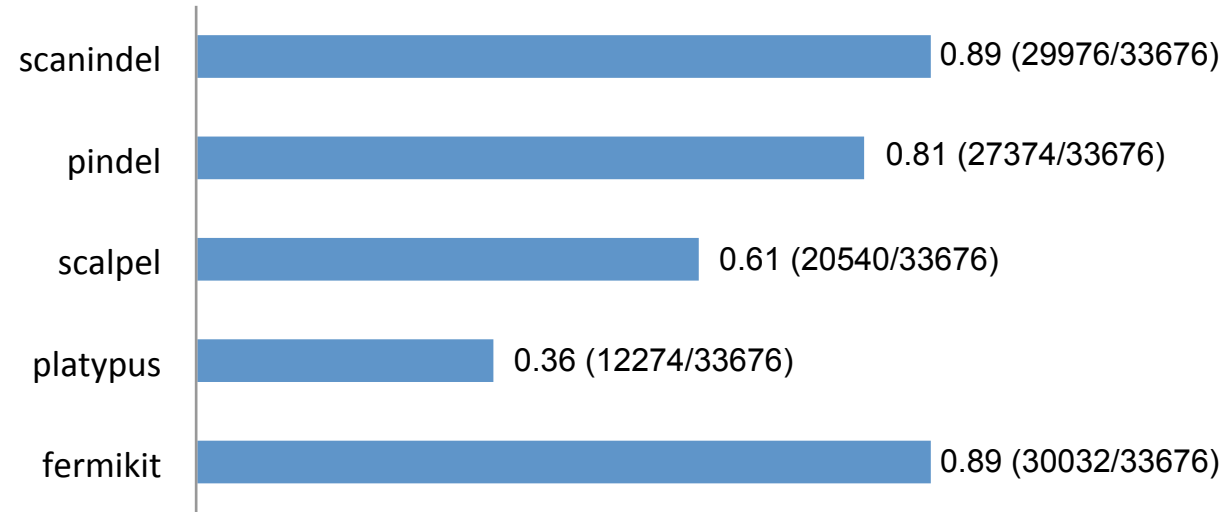

## Precision

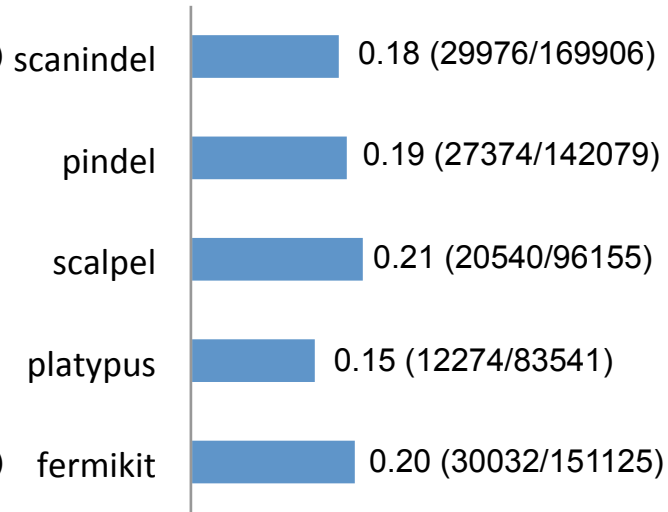

Indel 1 to 20bp

Supplement: Additional file 7: Figure S3. — Performance comparison for indel detection from short tandem repeat region of the NA12878 sample. Recall and precision are evaluated for ScanIndel, Pindel, Platypus, Scalpel, Platypus and FermiKit against the called indels from the Genome in a Bottle benchmark set for indels less than 20 bp. (PDF 31 kb) [file 13073_2015_251_MOESM7_ESM.pdf]
